# Supplementary figures and images for: SparsePro: An efficient fine-mapping method integrating summary statistics and functional annotations
Source: PLoS Genet. 2023 Dec 28;19(12):e1011104. doi: 10.1371/journal.pgen.1011104 (PMC10781022; doi:10.1371/journal.pgen.1011104)

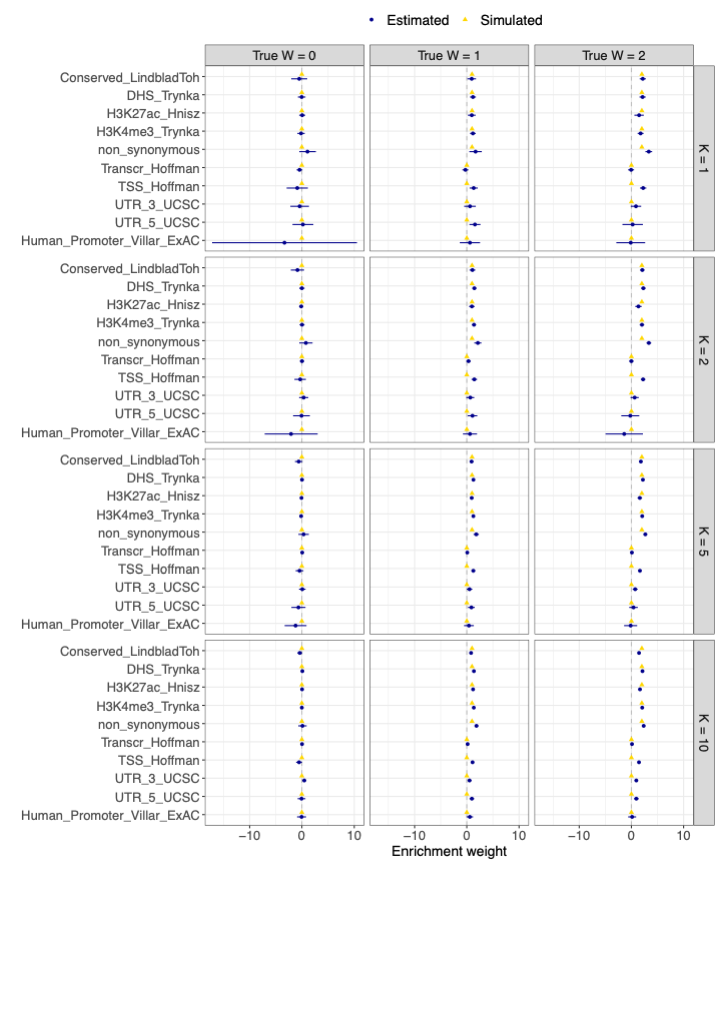

Supplement: S1 Fig — Each grid corresponds to a different simulation setting of K (number of causal variants) and W (enrichment intensity). Error bars represent 95% confidence intervals for enrichment estimates. Blue dots are estimated values and yellow triangles are simulated values. (TIFF) [file pgen.1011104.s021.tiff]

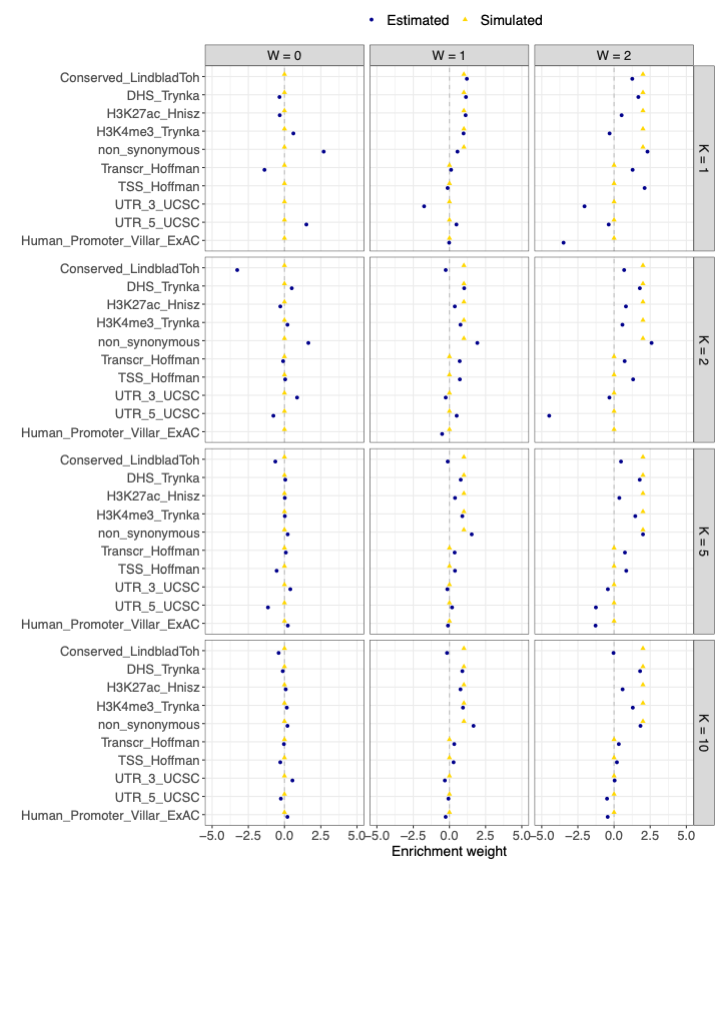

Supplement: S2 Fig — Each grid corresponds to a different simulation setting of K (number of causal variants) and W (enrichment intensity). PAINTOR does not provide confidence intervals for enrichment weights. Blue dots are estimated values and yellow triangles are simulated values. (TIFF) [file pgen.1011104.s022.tiff]

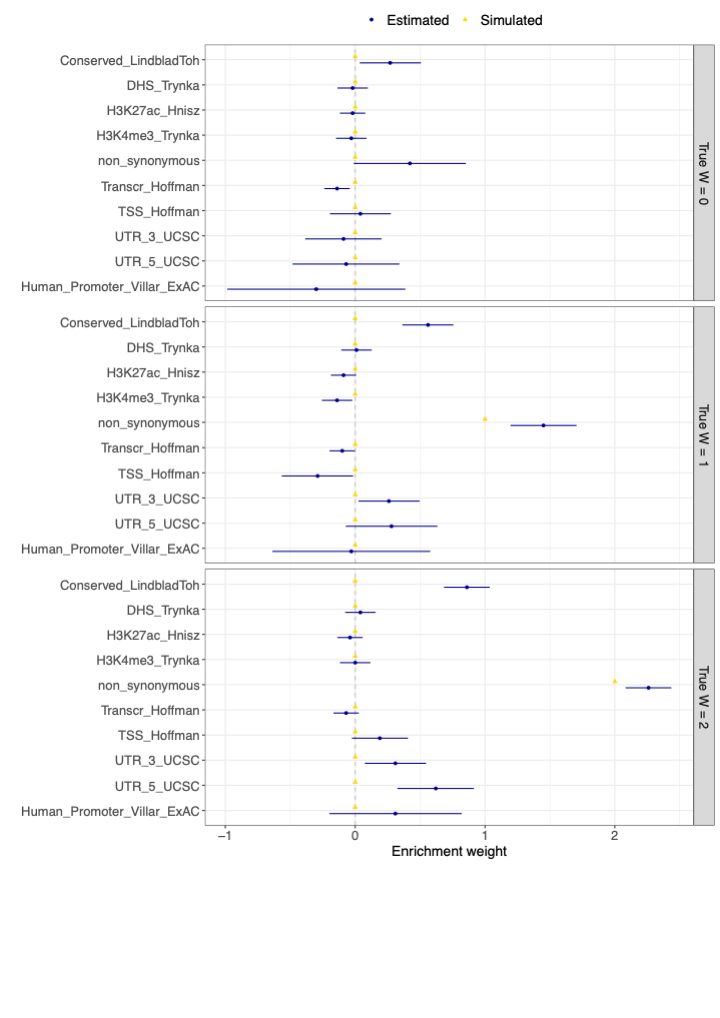

Supplement: S3 Fig — Each row represents a different simulation setting with W (enrichment intensity) = 0, 1, or 2. Error bars represent 95% confidence intervals for enrichment estimates. Blue dots are estimated values and yellow triangles are simulated values. (TIFF) [file pgen.1011104.s023.tiff]

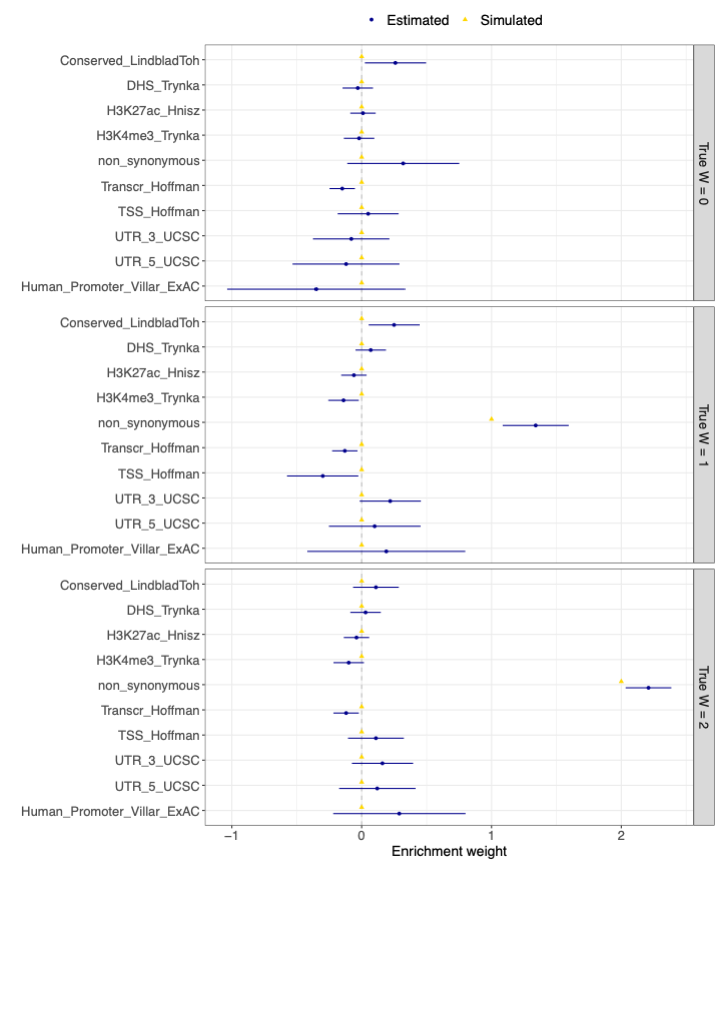

Supplement: S4 Fig — Each row represents a different simulation setting with W (enrichment intensity) = 0, 1, or 2. Error bars represent 95% confidence intervals for enrichment estimates. Blue dots are estimated values and yellow dots are simulated values. (TIFF) [file pgen.1011104.s024.tiff]

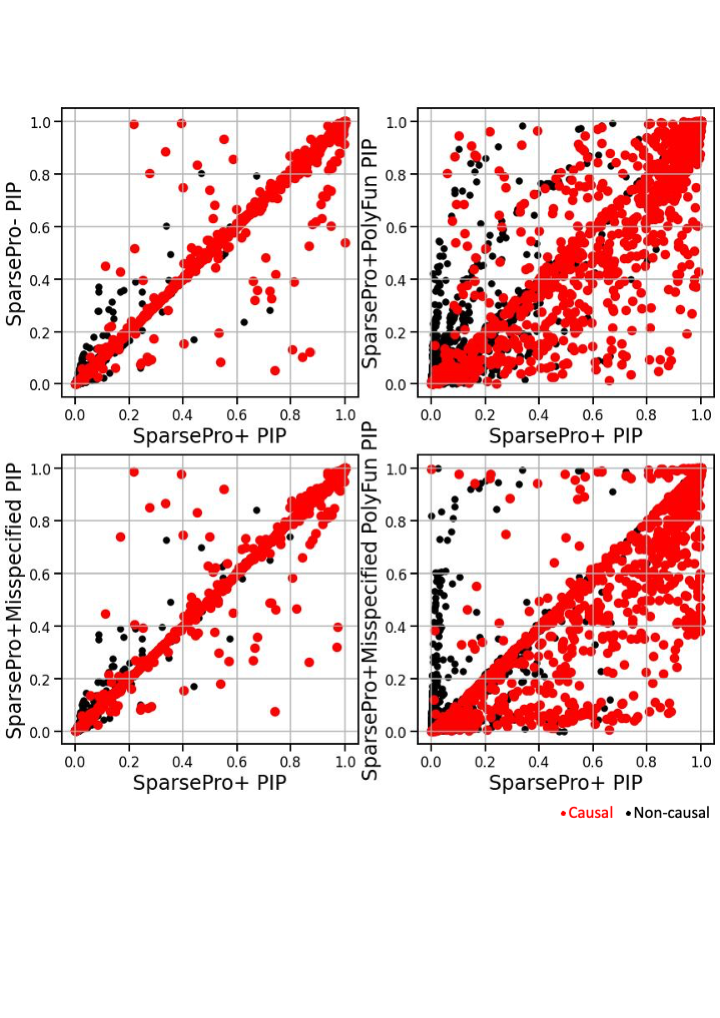

Supplement: S5 Fig — True causal variants are colored red and non-causal variants are colored black. (TIFF) [file pgen.1011104.s025.tiff]

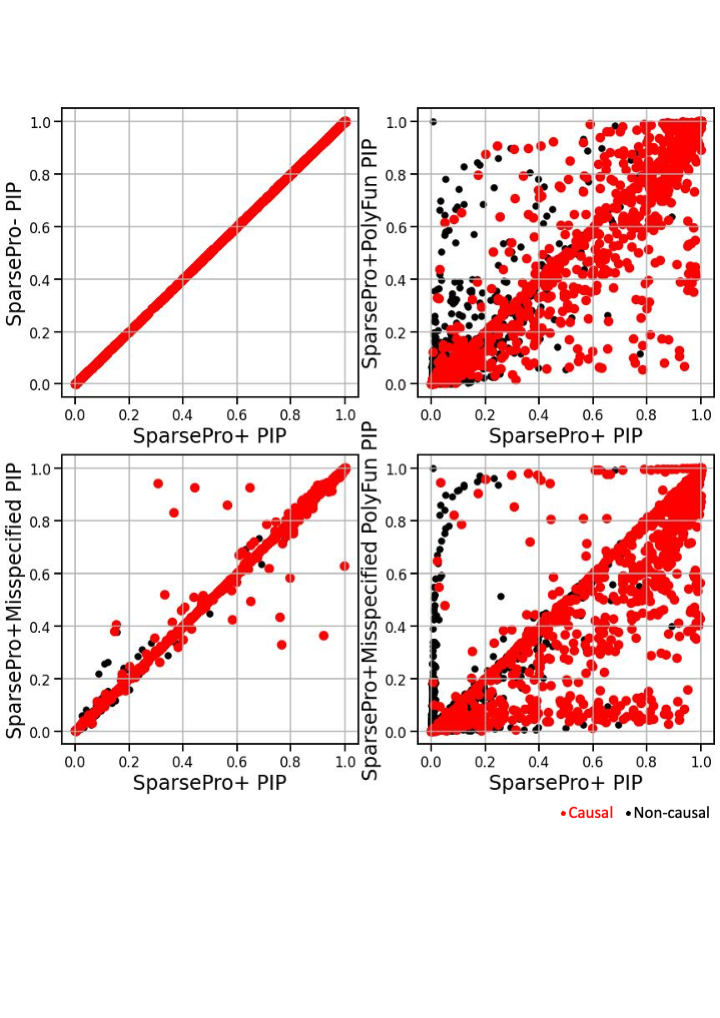

Supplement: S6 Fig — True causal variants are colored red and non-causal variants are colored black. (TIFF) [file pgen.1011104.s026.tiff]

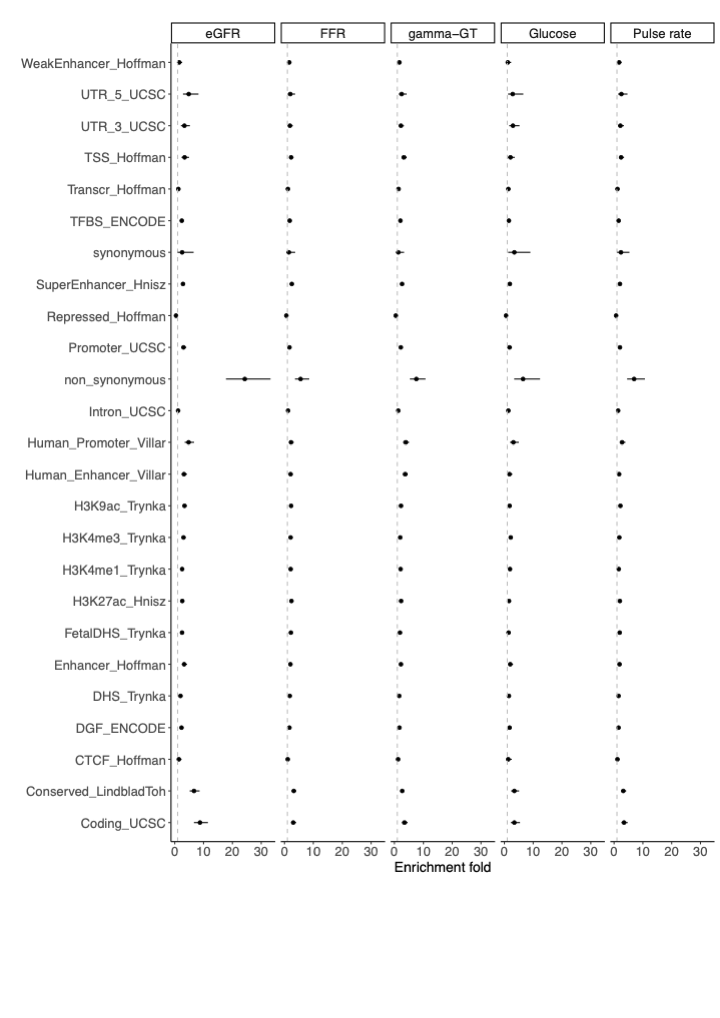

Supplement: S7 Fig — Each row denotes an annotation and each column denotes a functional biomarker. Error bars represent 95% confidence intervals for enrichment estimates. (TIFF) [file pgen.1011104.s027.tiff]

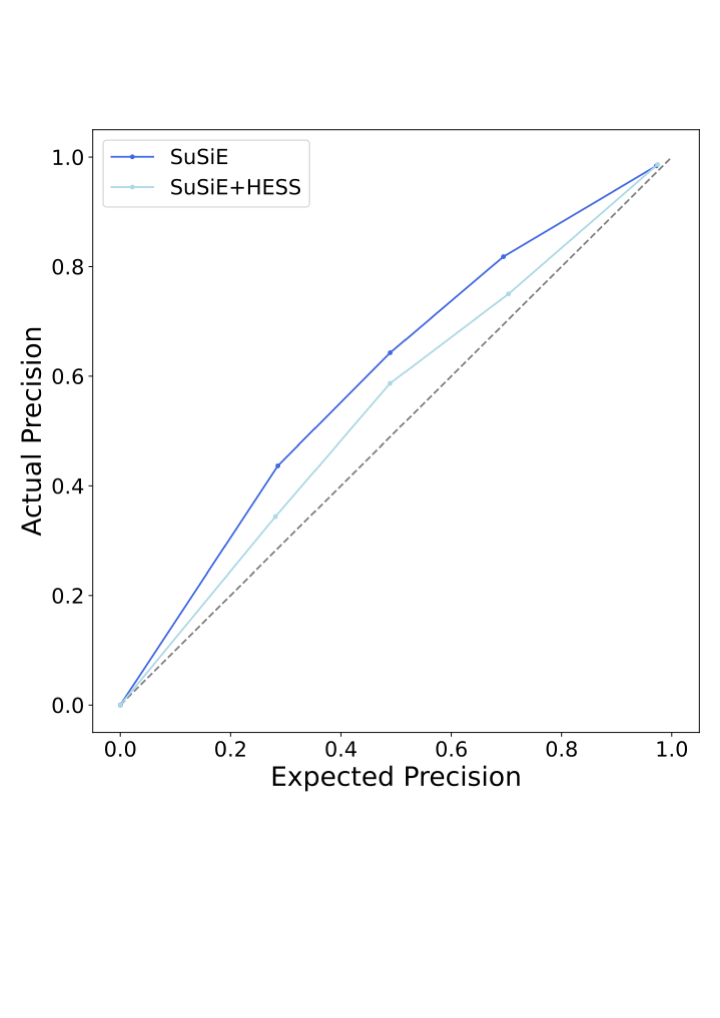

Supplement: S8 Fig — Variants are grouped into five bins according to their PIP values. Each dot represents one bin. The actual precision (y-axis) is plotted against the expected precision (x-axis) calculated by mean PIP values across all variants in the bin. (TIFF) [file pgen.1011104.s028.tiff]

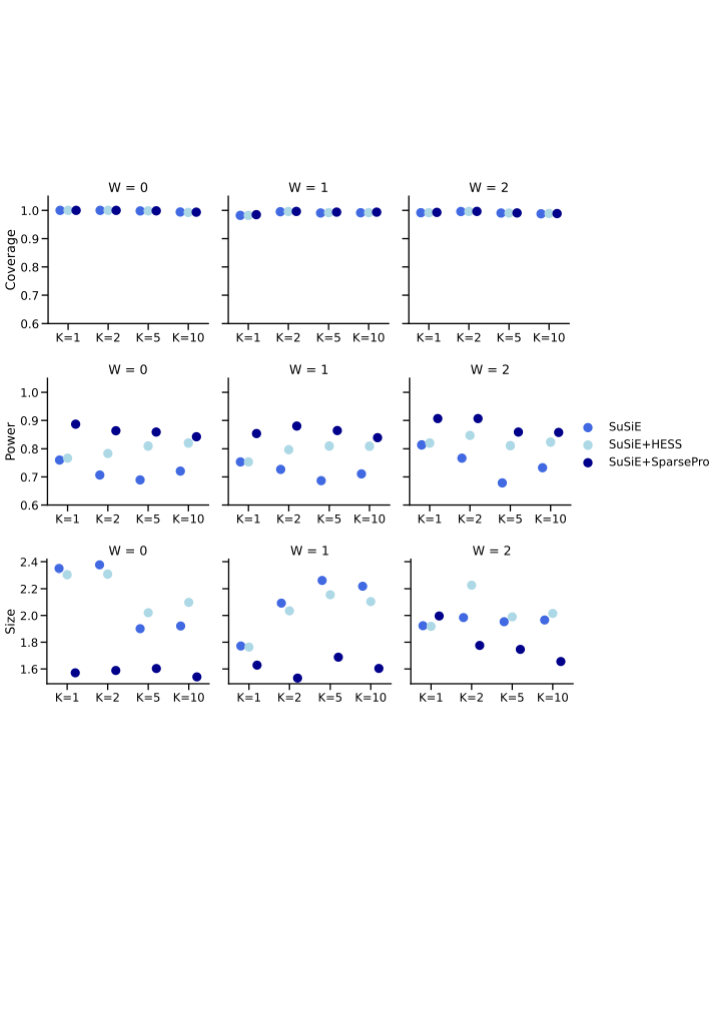

Supplement: S9 Fig — (TIFF) [file pgen.1011104.s029.tiff]

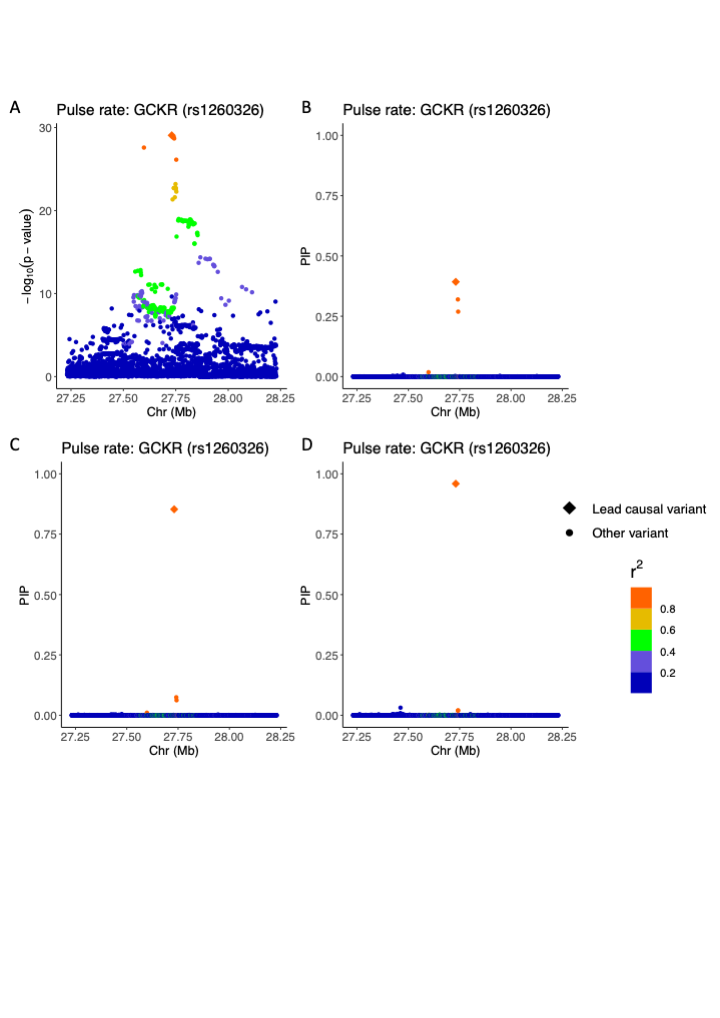

Supplement: S10 Fig — (A) GWAS summary statistics for pulse rate at the GCKR locus. (B) Fine-mapping results from SparsePro-. (C) Fine-mapping results from SparsePro+. (D) Fine-mapping results from SparsePro+PolyFun. P-values from GWAS and inferred posterior inclusion probabilities from fine-mapping are illustrated. Variants within a ±500kb window are colored by their linkage disequilibrium r2 with rs1260326. (TIFF) [file pgen.1011104.s030.tiff]

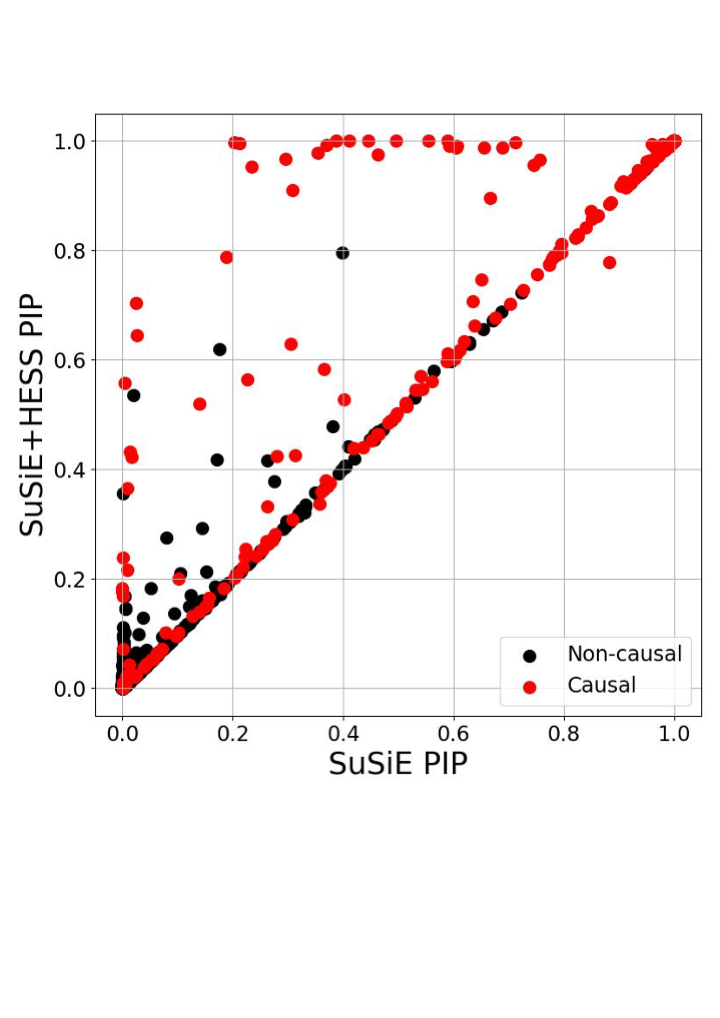

Supplement: S11 Fig — True causal variants are colored red and non-causal variants are colored black. (TIFF) [file pgen.1011104.s031.tiff]

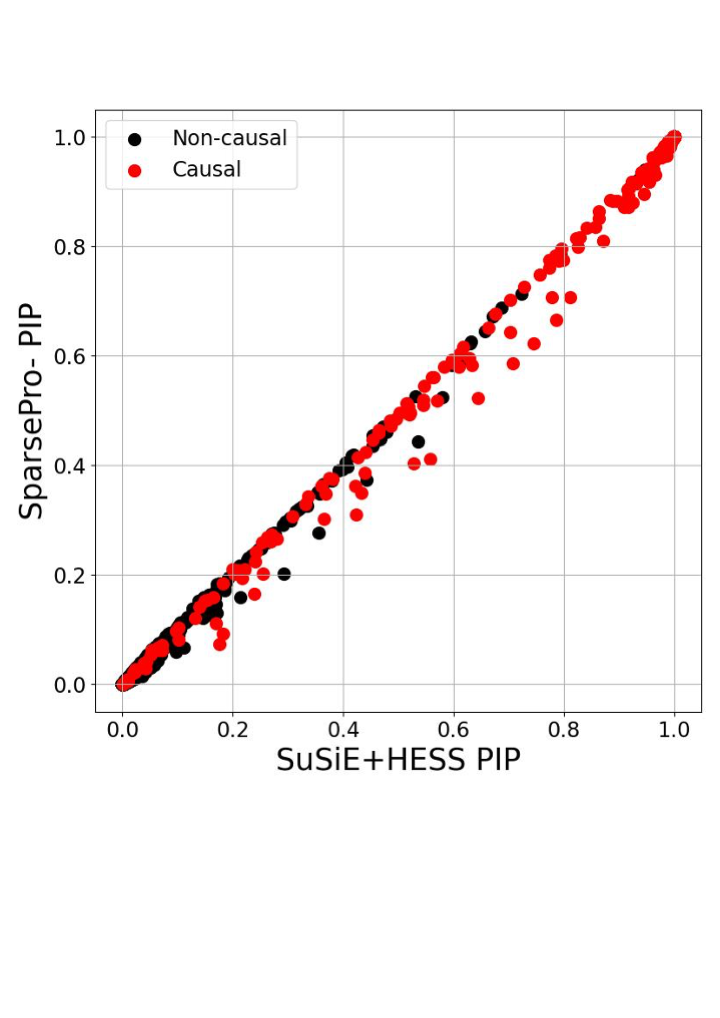

Supplement: S12 Fig — True causal variants are colored red and non-causal variants are colored black. (TIFF) [file pgen.1011104.s032.tiff]

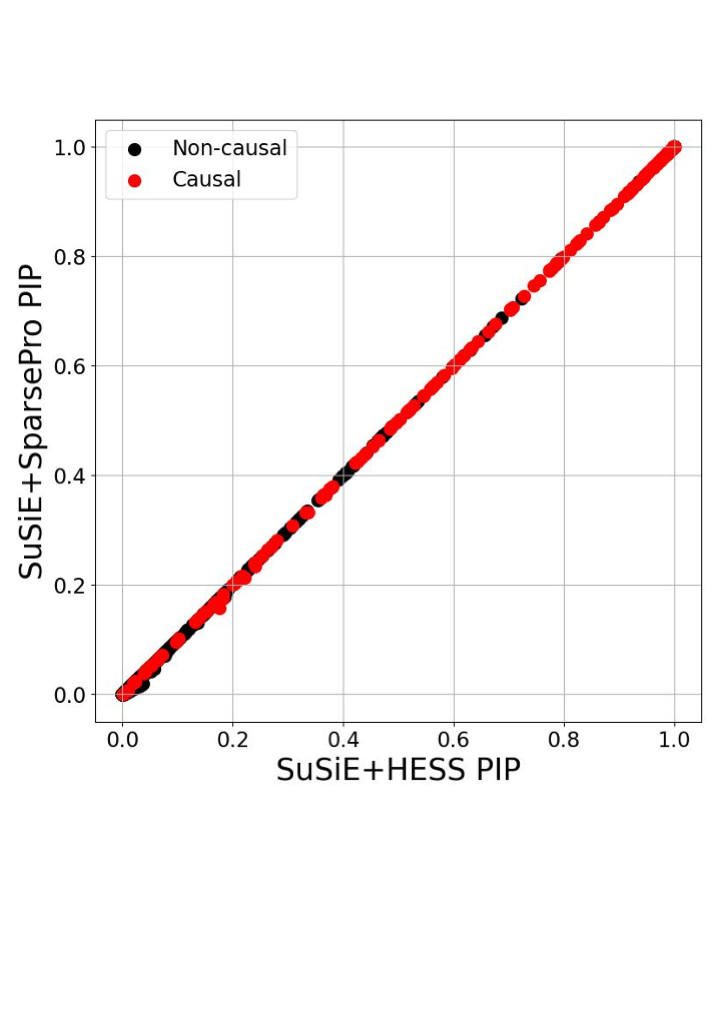

Supplement: S13 Fig — True causal variants are colored red and non-causal variants are colored black. (TIFF) [file pgen.1011104.s033.tiff]
