# Supplementary material for: SparsePro: An efficient fine-mapping method integrating summary statistics and functional annotations
Source: PLoS Genet. 2023 Dec 28;19(12):e1011104. doi: 10.1371/journal.pgen.1011104 (PMC10781022; doi:10.1371/journal.pgen.1011104)
Supplement: Attachment — Submitted filename: sparsepro_review.pdf [file pgen.1011104.s034.pdf]

SparsePro Manuscript Comments

The authors propose a method for estimating the hyperparameters of the sum of single effects regression. In particular they leverage functional annotation enrichments to specify informative prior inclusion probabilities for different variants, and leverage heritability estimates to set the effect size and residual variance hyper-parameters. By incorporating this enrichment information they are able to demonstrate improvement over fine-mapping methods that either do not leverage functional annotations, or leverage functional annotations through different means (e.g. Polyfun, which computes prior inculsion probabilities given partitioned heritability estimates across a set of annotations). These are important contributions, as the selection of these hyperparameters can greatly influence the calibration and power of finemapping.

While I am generally positive about the work put forth here, my main reccomendations are to focus the discussion and commentary on the benefits of including functional annotations, and providing more clear rationale for the use of heritability information to set the effect and error variance hyperparameters. The authors should modify their discussion of the algorithmic differences between SuSiE and SparsePro because they do not seem accurate-- as far as I can tell, for a fixed set of hyperparameters (prior inclusion probability, effect variance, residual variance) the coordinate ascent variational inference (CAVI) employed for SparsePro and SuSiE's IBSS algorithm (which is also CAVI) are the same.

Estimating prior inclusion probabilities (Sparspro vs Polyfun) To my understand, Polyfun provides a heuristic for forming the prior inclusion probabilites based on a heritability partition. Sparsepro takes a less heuristic approach by directly estimating enrichment of selected variants, and using those enrichments to refine the posterior approximations made by SuSiE. I very much support this approach, and the authors successfully show through extensive simulations how their method improves on the heursitic approach to estimating the prior inclusion probabilities developed in Polyfun.

Estimating variance hyperparametes (Sparsepro vs SuSiE) SuSiE uses a variational empricial Bayes approach to estimate the effect variance and residual variance-- this just means optimizing the objective w.r.t to these hyperparameters. In contrast, Sparsepro fixes these hyperparemeters to values informed by heritability estimates. For example, the residual variance is set to 1-h2.

The residual variance is fixed to 1 - h2 where h2 is a locus level heritability estimate. In contrast, a conservative approach would be to set the residual variance to 1. I'm concerned that setting the residual variance to 1-h2 may disrupt calibration of the posterior. Basically,

while h2 is an estimate of the heritability in the locus, finemapped association signals will only explain a portion of this heritability. 1-h2 may be too small, and encourage the model to select variants in a way that is anti-conservative.

The variational approximations are identical The discussion and supplemental materials emphasize the differences in computation between SuSiE's IBSS algorithm, and the variational updates derived in this paper. However, it is important to note that the variational approximation for Sparsepro and SuSiE are identical $q(\beta, S) = \prod_k q(\beta_k, s_k)$. Consequently all differences in performance between SuSiE and Sparsepro- (without annotation) should be explained by (1) differences in the hyperparameters/hyperparameter estimation procedure and (2) implimentation details (e.g. convergence criteria, order of coordinate updates, etc., which may influence which local optima of the variational objective is found).

In particular the following does not seem correct 214:215 "In SuSiE, Bayes factors were normalized by sum of Bayes factors across all variants while SparsePro uses the softmax function to normalize posterior probabilities which increased power for identifying causal variants". I believe the marginal log Bayes factors are equal (up to a constant) to the posterior (log) probabilities referenced here (th 4th expression in supplementary material, page 27). Thus normalizing Bayes factors is equivalent to applying softmax of the log probabilities.

Suggested Revisions

- Clarify the similarities and differences between Sparsepro and SuSiE. I believe the variational approximations are the same, but the real contribution here are annotation and heritability informed hyperparameter settings, which are an important contribution that can stand on there own.
- Sparsepro- and SuSiE shoud be identical up the the setting of the effect variance and residual variance hyperparameters. Commentary attempting to explain the difference in performance between SuSiE and Sparsepo- should be revised, because at times it implies a difference in the algorithm/optimization procedure which does not seem to be correct.
- Please discuss/justify the heritability based estimates for effect variance and residual variance. In particular I am concerned that useing 1-h2 fro residual variance will make the algorithm anticonservate (see above) by underestimating the residual variance in the regression problem.
- Assess the calibration of PIPs for Sparsepro+ (e.g. Figure S1 in SuSiE manuscript)-- the AUC plots tell us that ranking variants by PIP is good, but it doesn't tell us that thresholding at some nominal PIP value controls the false positive rate. Good PIP callibration would go a long way in addressing my conerns about the choice of residual variance parameter.

Minor points

- Maybe a simpler enrichment analysis for the UKBB biomarkers would be (1) causal variants in this phenotype vs (2) causal variants discovered in other phenotypes. It would more clearly highlight that the enrichment of the tissue-specific annotation in the relvant biomarker is above and beyond the background level enrichment of enrichment across causal variants discovered in all phenotypes.
- Were causal variants defined as the top variant per credible set or all variants in the credible set?
- It is not clear to me which annotations are used in the UKBB biomarkers analysis. This should be clearly stated in 4.4 or methods.
- To clarify the comparison between polyfun and Sparsepro it may be good to (1) run Sparsepro with the prior inclusion probabilities derived from polyfun and (2) fit Sparsepro with the exact same annotations used in polyfun (without screening annotations based on significance first). (1) vs Sparsepro+ would demonstrate that Sparsepro+ is making better use of the annotation information. (2) vs Sparsepro+ would emphasize the benfit of selecting annotations based on Gtest.
