# Supplementary material for: SparsePro: An efficient fine-mapping method integrating summary statistics and functional annotations
Source: PLoS Genet. 2023 Dec 28;19(12):e1011104. doi: 10.1371/journal.pgen.1011104 (PMC10781022; doi:10.1371/journal.pgen.1011104)
Supplement: Attachment — Submitted filename: plos_genetics_response_letter_20230628.pdf [file pgen.1011104.s035.pdf]

Dear Dr. Wang and Dr. Zhu,

We thank you and the reviewers for considering our manuscript titled ***"SparsePro: an efficient fine-mapping method integrating summary statistics and functional annotations"***, and providing valuable comments and suggestions that have helped us to significantly improve the manuscript. In response to the reviewers' comments, we made substantial improvements to the manuscript based on their suggestions. In particular, we have added the following new sections in our revised manuscript.

First, we explicitly connected our proposed approach with SuSiE and established equivalence between the SuSiE IBSS algorithm and a well-studied paired mean field variational inference algorithm [2] in the **Equivalence between the SuSiE IBSS algorithm and a paired mean field variational inference algorithm** section in lines 8-52 of the Supplementary Notes. We hope these connections can provide a better understanding of the efficiency and accuracy of both algorithms.

Second, we explained our motivation for estimating hyperparameters with local heritability estimates in the **Hyperparameter estimation** section in lines 53-72 of the Supplementary Notes. Specifically, local heritability estimates can reduce the number of parameters to be estimated by fine-mapping algorithms, resulting in improved accuracy and efficiency. Additionally, we incorporated this strategy in SuSiE and observed improved power for fine-mapping compared to its original implementation.

Third, we clarified our attainable coverage-based strategy for posterior summaries in the **Posterior summaries** section in lines 73-112 of the Supplementary Notes. Specifically, we calculated attainable coverage for each effect group and only effect groups with attainable coverage greater than ρ were summarized to ρ-level credible sets. Similarly, we applied this strategy to SuSiE and proved its benefits over the original purity-based implementation.

We posit that these three aspects of SparsePro represent important contributions that extend the capabilities of SuSiE, and we have expanded the **Discussion** section in lines 218-238 accordingly.

Fourth, following the reviewers' suggestions, we modified our genome-wide simulations to directly compare methods for deriving functionally-informed priors and investigate the impact of annotation misspecifications as detailed in the **Methods** section in lines 344-362. We showed that SparsePro is more effective and robust than PolyFun in the **Results** section in lines 129-176.

Fifth, we expanded discussion on the utility of functional annotations in fine-mapping and the potential benefits of the G-test in the **Discussion** section in lines 239-263. Through real data examples, we demonstrated the “non-synonymous” annotation was useful in prioritizing causal variants when statistical evidence alone was not able to distinguish causal variants from other correlated variants.

We believe that these additional analyses, modifications, and clarifications have significantly improved the quality of our manuscript. We have described other modifications based on reviewers’ suggestions in the enclosed point-by-point response letter.

We thank you again for your consideration and look forward to hearing from you!

Sincerely,

Wenmin Zhang, on behalf of all authors

Quantitative Life Sciences Program, McGill University

Response to Reviewers

Reviewer 1:

Zhang et al present their extension of the SuSiE model to incorporate functional annotations. SparsePro prevents irrelevant annotations from contaminating the model by testing the functional annotations before integrating them into the model. It estimates the enrichment coefficients within the IBSS algorithm. Moreover, it estimates some hyper-parameters outside the IBSS algorithm to avoid convergence issue. The manuscript presents simulation results to support the method. They also show application result on UKBiobank traits. It is well written. As SuSiE becomes a popular approach for fine-mapping, it is desirable to have a version supporting functional annotations.

We thank the reviewers for their supportive comments.

Major comments:

1. The SparsePro model is essentially the SuSiE model with functional annotations, with some changes to the prior and residual variance estimation. As most readers are familiar with SuSiE, I suggest using the terminology and concepts from SuSiE in the manuscript. For example, replacing 'effect group' with 'credible set'; using similar credible set definition as in SuSiE. The derivations in supplementary section 1 before annotation estimation are same as in SuSiE, but the SuSiE paper is not cited.

We appreciate this comment. We have now clarified our connections with SuSiE, especially the equivalence between the SuSiE IBSS algorithm and the paired mean field variational inference algorithm as detailed in the **Equivalence between the SuSiE IBSS algorithm and a paired mean field variational inference algorithm** section in lines 8-52 of the Supplementary Notes. We added citations for the SuSiE paper in lines 33-34, lines 40-41 and lines 49-52 of the Supplementary Notes. We changed our previous definition of causal set to a similar credible set definition as in SuSiE and proposed an attainable coverage-based strategy for summarizing posterior probabilities as detailed in the **Posterior summaries** section in lines 73-112 of the Supplementary Notes. We find that the concept of effect groups is important for illustrating the dimensionality reduction perspective as explained in lines 10-18 of the Supplementary Notes. It is also difficult to replace effect groups to credible sets without loss of clarity, as only

effect groups with enough statistical support can be summarized into credible sets. Additionally, we proposed effective strategies for estimating hyperparameters as detailed in the **Hyperparameter estimation** section in lines 53-72 of the Supplementary Notes. We have included additional comparisons of SparsePro vs. SuSiE, as well as the original implementation of SuSiE vs. SuSiE with our proposed modifications to illustrate their utilities. We believe these aspects of SparsePro represent important contributions that extend the capabilities of SuSiE, as discussed in lines 214-234 of the **Discussion** section.

2. The statement on line 214 said 'in SuSiE, Bayes factors were normalized by sum of Bayes factors ... which increased power for identifying causal variants.' This statement is not accurate. The softmax is same as normalizing weighted Bayes Factors (weighted by the prior probability π_g). So the log probability in page 37 3rd equation is same as $\log(\pi_g *$ Bayes Factor$)$. This is not the cause for the SparsePro higher power in the simulation. The higher power from SparsePro- may caused by estimating hyperparameters outside the iterative algorithm, and different PIP definition.

We thank the reviewer for pointing out this equivalence. We have now removed the inaccurate statement and added the **Equivalence between the SuSiE IBSS algorithm and the paired mean field variational inference algorithm** section in lines 8-52 of the Supplementary Notes to demonstrate the equivalence. We also have included comparisons between the original implementation of SuSiE and SuSiE applying our proposed strategies for estimating hyperparameters and summarizing posterior probabilities as detailed in the **Hyperparameter estimation** section in lines 53-72 of the Supplementary Notes and the **Posterior summaries** section in lines 73-112 of the Supplementary Notes. These comparisons confirmed that our proposed strategies can substantially improve the power of both SparsePro and SuSiE.

3. The PIP for each variant is computed as max among groups. Why not computing using $1-\Pi_k(1-\gamma_{kg})$, which is the theoretical definition of PIP?

We have now clarified the connection between our maximization-based approach and the multiplication-based approach adopted in SuSiE in the **Posterior summaries** section in lines 73-112 of the Supplementary Notes. Specifically, the maximization-based approach takes advantage of the fact that a variant can only contribute informatively to at most one effect group as explained in lines 89-93 of the Supplementary Notes. The maximization-based approach is equivalent to the multiplication-based approach used in

SuSiE when the negligible posterior probabilities are disregarded. As in line 108 of the Supplementary Notes, we have:

$$PIP_g = 1 - \Pi_k(1 - \gamma_{kg}) = 1 - (1 - \max(\gamma_{1g}, \ldots, \gamma_{Kg})) = \max(\gamma^*_{1g}, \ldots, \gamma^*_{Kg})$$

where

$$\gamma_{kg} = \begin{cases} \gamma^*_{kg}, & \text{if } \gamma^*_{kg} = \max(\gamma^*_{1g}, \ldots, \gamma^*_{Kg}) \\ 0, & \text{otherwise} \end{cases}$$

As demonstrated in **Supplementary Figure** S13, despite its simplicity, the maximization-based approach yielded similar PIP to the multiplication-based approach.

4. From line 310-313, it seems that the 95% causal set (1 set containing all causal variants) is used in the set-level comparisons, not 95% credible sets as defined in SuSiE. Is there any reason not using credible sets for comparisons? SparsePro, SuSiE and FINEMAP all output 95% credible sets. It is unnecessary to combine them into one causal set. I suggest conducting set-level comparisons using 95% credible sets (coverage / power / size of each single 95% credible set).

We appreciate this suggestion. We have now directly conducted set-level comparisons between SparsePro and SuSiE, although we could not find an approach to derive comparable sets based on the results from FINEMAP. In lines 100-112 of the **Results** section, **Figure** 3A and **Supplementary Table** S1, we show that SparsePro was able to generate 95% credible sets with a similar coverage and a higher power at a smaller size. We also applied our strategy for summarizing posterior probabilities to SuSiE and proved its benefits over the original purity-based implementation in the **Posterior summaries** section in lines 101-105 of the Supplementary Notes.

5. The method estimates the enrichment coefficient using 'one-at-a-time' coordinate ascent. Is this same as jointly estimating the coefficients? Is it possible to estimate them jointly? For your reference, TORUS (Wen, X., 2016.) estimates the coefficients jointly using EM.

We thank the reviewer for this question. Yes, we jointly estimated the enrichment weights for all annotations included in the model. In the closed form updates for the mth annotation, we accounted for the effect of other annotations with $softmax(\sum_{m' \neq m} A_{gm'} w_{m'})$, as demonstrated in line 131 of the Sup-

plementary Notes. Through iterating over all annotations, upon convergence, we obtain a joint estimate of the enrichment weight vector $\mathbf{w}$ that can be used to derive functionally-informed priors as described in lines 134-135 of the Supplementary Notes. Updating all enrichment weights in one iteration can be achieved by gradient-based methods but they are more computationally expensive compared to the closed form updates. Additionally, we also separated the description for the univariate G-test and the derivation of the joint estimates of enrichment weights into two sections in the Supplementary Notes to avoid confusion.

Minor comments:

1. The credible sets from SuSiE has a purity > 0.5 filter by default. Is the same purity filter applied in other methods? If not, the comparison is unfair.

We have now clarified our approach for posterior summaries in the **Posterior summaries** section in lines 73-112 of the Supplementary Notes. We explained our motivation for replacing the purity-based filtering with attainable coverage-based summaries. We demonstrated that our proposed approach yielded better set-level summaries than using purity > 0.5, as 95% credible sets derived from the same SuSiE-inferred posterior probabilities using our approach achieved a comparable coverage, a higher power and a smaller size as demonstrated in **Supplementary Figure** S9 and detailed in lines 101-105 of the Supplementary Notes.

2. The simulation has a per-variant heritability 10^{-4}. Does this mean '–simu-hsq' is $K * 10^{-4}$ in GCTA simulation? Or does it mean the simulated causal variants have similar effect size?

We apologize for this confusion. We have now clarified in lines 308-309:

Then, we used the GCTA GWAS simulation pipeline [4] to simulate a continuous trait with a total heritability of $K \times 10^{-4}$.

3. The relationship between the simulation parameter W and the relative enrichment vector w in section 4.1 page 10 is unclear.

We apologize for this confusion. We have now renamed the simulation parameter W as enrichment intensity to help clarify the relationship. Specifically, in the simulated enrichment vector $\mathbf{w}$, the entries for

annotations designated to be enriched had a value of W, while for other irrelevant annotations, the entries are 0. We added a column of “Simulated w” in **Supplementary Tables** S3, S5, S6 and S7 and yellow triangles in **Supplementary Figures** S1-4. We have also added text in lines 306-307 to clarify:

In the simulated weight vector $\mathbf{w}$, the entries that correspond to the these enriched annotations had a value of W.

4. Line 242 ’causal effect sizes may vary across different subpopulations’, where does the subpopulation come from? This is just single study fine-mapping.

We apologize for this confusion. This sentence was meant to discuss possible future directions, such as fine-mapping in diverse populations. We removed the sentence to avoid confusion.

5. Do the computational times in figures include estimating τ_β, τ_y and testing of functional annotations?

Yes, the computation time does include steps for estimating parameters and testing for enrichment. We have now clarified it in lines 323-324:

Computation time was recorded on a 2.1 GHz CPU for fine-mapping programs including all procedures.

6. Any reason to use log20 for entropy difference cutoff? Any reason to use 10^{-5} p value threshold for G-test?

We use the entropy cutoff to account for scenarios where there are no causal variants in a fine-mapped locus. As a result, in such cases, the posterior distributions of the sparse indicators will be very close to their prior distributions, having a high entropy. In contrast, when there is a causal signal, the posterior distribution will have a low entropy with probability mass concentrating on a few variants in high correlation with the causal variant. A default cutoff of $\log 20$ corresponds to a upper limit of 20 variants in tight LD with each other in a legitimate credible set. Users can adjust this value based on the level of LD tightness observed in their genotype data as detailed in lines 94-100 of the Supplementary Notes.

For G-test, in our experience, a p-value threshold of 10^{-5} was able to select annotations such that the model retains sufficient amount of information while maintaining interpretability. Users can also adjust this threshold based on their preference and contexts. In SparsePro, selection of annotations based on G-test does not have a dramatic impact on fine-mapping results as demonstrated in **Supplementary**

Tables S2 and S8. This is because when irrelevant annotations are included in the joint estimates, their estimated enrichment weights are typically small as demonstrated in **Supplementary Table** S6 and discussed in lines 245-258, thus having a limited impact on the derived functionally-informed priors.

7. In the genome-wide simulation, the results in the 1-MB center of each 3-MB window are considered. But the signal could also at/close to the 1-MB center boundary. How to analyze these signals?

In our sliding window approach, we included all variants within each 3-Mb sliding window for fine-mapping. This means that each 1-Mb region is fine-mapped three times. However, we only retained the fine-mapping results for the central 1-Mb region. This approach allowed us to fine-map variants together with neighboring variants within at least 1-Mb, which helped to mitigate boundary effect. If a variant was located in the left border, its fine-mapping result was obtained from the preceding window. Similarly, if it was located in the right border, its fine-mapping result was obtained from the succeeding window. Fine-mapping result of a variant was only obtained from the current window if it was located within the central 1-MB region. We have added descriptions in lines 351-355:

In these fine-mapping analyses, variants in each 3-Mb sliding window were fine-mapped jointly but results were only retained for variants located in the 1-Mb region central to the window. We obtained genome-wide fine-mapping results by concatenating the results from these 1-Mb central regions. Therefore, variants were fine-mapped together with neighboring variants within at least 1-Mb to mitigate boundary effect.

8. What's the coefficient w scale in Fig S7?

We apologize for this confusion. The original Figure S7 is the plot for coefficients estimated by PolyFun using LD score regression. These coefficients are not equivalent to the estimates of enrichment weights as our estimated w. We have now detailed these statistics in **Supplementary Table** S7.

Reviewer 2:

Zhang et al. proposed an interesting enhancement of the SuSiE model proposed by Wang et al. JRSSB 2021 to perform informed fine-mapping using side information/annotation. The authors show some convincing evidence that their approach (SparsePro) has greater power than some competing methods, such as SuSiE+Polyfun. Overall the approach is sound, and I am mostly positive regarding the manuscript. The level of detail provided by the authors is satisfactory. Still, some typos are disrupting the overall quality of the manuscript as well as some statements that need to be clarified.

We thank the reviewers for their supportive comments.

Major comment:

1) While most of the manuscript reads quite well, there is a couple of sentences that do not flow well or are grammatically incorrect, e.g., in the last paragraph of the introduction, the authors wrote "In line with the idea of grouping correlated variants together into effect groups, we proposed Sparse Projections to Causal Effects (SparsePro) to further improve fine-mapping efficiency and accuracy. First, within each effect group, we additionally incorporate" in this case, first needs to be followed by a sentence with an active verb before additionally. I am not a native English speaker, and I am aware that it can be hard to draft a manuscript. However, before considering the manuscript for acceptance, the authors need to proofread the manuscript to correct some of these problematic sentences.

We thank the reviewer for this suggestion. We have proofread our manuscript and corrected these grammatical errors. We believe the readability of our manuscript has substantially improved.

2) The authors wrote: " Second, we use an efficient variational inference algorithm to further simplify the intuitive algorithm proposed in SuSiE and improve computation efficiency." I have had a close look at the algorithm proposed by Zhang. While the author does not explicitly compute the marginal Bayes factor for each variant, given that we do not use any annotation, the coordinate ascend seems very similar to the one proposed by Wang et al. As Wang and colleague provided the complexity of each coordinate ascend update, I think it would be interesting that the authors provide the complexity of each coordinate ascend update of SparsePro. This would make it more explicit that the gain in computational speed is not only due to the implementation. Because for the moment, it is not clear to me how this approach differs

from the IBSS. Perhaps the author could elaborate on the computational complexity of their VA of the Single Effect Model proposed by Wang et al.

We thank the reviewer for pointing out the equivalence. We have now removed the incorrect discussion and clarified the equivalence in the **Equivalence between the SuSiE IBSS algorithm and a paired mean field variational inference algorithm** section in lines 8-52 of the Supplementary Notes.

3) I would be interested in seeing a set of simulations in which the annotations are misspecified/measured with noise and potential bias toward non-causal SNPs. While substantial efforts are made to get high-quality annotations, it is not unlikely that many of those are poorly measured or biased. I would be interested in seeing some simulations in which the author would consider poorly measured annotations and see if that could generate some low-coverage credible set. In general, my overall question is that given that you have some annotations, is it worth it to include them in a fine mapping procedure, or could that potentially ”harm” your results. Could you generate a new set of simulations in which annotations are measured with noise? Furthermore, could try to come up with a set of simulations that could lead to problematic coverage due to annotations. For example, suppose that you use the following annotation (that is made to be problematic) in a case where you consider a model with K SNP. Consider the following K annotation for each non-casual SNP set annotation k to its correlation with causal SNP k and for the causal SNP set each of their K annotations by sampling a random number 0 and 1

We thank the reviewer for raising the important issue of annotation misspecification and measurement errors. We have now modified our genome-wide simulations to investigate the impact of annotation measurement errors as detailed in the **Methods** section in lines 344-362. Following the reviewer's suggestion, we used the “conserved sequence” as a surrogate for the simulated enriched “non-synonymous” annotation. These two annotations partially overlap, hence the former can serve as an annotation measured with errors. We showed that SparsePro is more robust than PolyFun in case of annotations measured with errors in the **Results** section in lines 129-176. We also discussed how estimating annotation enrichment weights before deriving functionally-informed priors can improve robustness for functional fine-mapping methods in the **Discussion** section in lines 241-245.

Minor comment:

1) There is a type after equation 2 in the supplement for the condition variational approximation. It is written $s_{kg=1}$ whereas it should be $s_{kg} = 1$; please go through the equation to correct the other typos

We thank the reviewer for noting this typo. We have made corrections.

2) The equation below, "Therefore, the posterior probability of the gth variant being causal in the kth effect group can be estimated as:" seems somewhat not correct. The input of in the softmax function is a scalar, whereas it should be a vector. The posterior probability of the gth variant being causal in the kth effect group should be the g component of this softmax.

We thank the reviewer for noting this inaccuracy. We have now avoided using the softmax function on scalars and the original equation has been editted in line 48 of the Supplementary Notes:

$$\gamma^*_{kg} := q(s_{kg} = 1, \mathbf{s}_{k\backslash g} = \mathbf{0}) = \frac{\exp(\log \tilde{\pi}_g - \frac{1}{2}\log \tau^*_{kg} + \frac{1}{2}\tau^*_{kg}\mu^{*2}_{kg}))}{\sum_{g'} \exp(\log \tilde{\pi}_{g'} - \frac{1}{2}\log \tau^*_{kg'} + \frac{1}{2}\tau^*_{kg}\mu^{*2}_{kg'}))}$$
$$= \frac{\tilde{\pi}_g \sqrt{\exp(\tau^*_{kg}\mu^{*2}_{kg})\tau^{*-1}_{kg}}}{\sum_{g'} \tilde{\pi}_{g'} \sqrt{\exp(\tau^*_{kg'}\mu^{*2}_{kg'})\tau^{*-1}_{kg'}}}$$

3) In the SuSiE-rss manuscript, Zou and colleagues spend a substantial amount of work dealing with problematic LD. I would be interested to explicitly say what is implemented in SparsePro to circumvent problems related to LD matrices that are not full rank or allele flipping problem.

We thank the reviewer for raising the important point on problematic LD. We have discussed issues related to LD rank deficiency and allele flipping in lines 265-271:

First, there are challenges related to allele flipping and LD rank deficiency when using summary statistics for fine-mapping. SparsePro, similar to Zou et al [5], does not require a full-rank LD matrix as it does not require matrix inversion throughout the algorithm. However, allele flipping can lead to algorithm convergence issues. To address this, it is recommended that users closely monitor the convergence of the algorithm and utilize scripts we provided to automate the formatting of GWAS summary statistics to match alleles in the LD reference panel. By taking these precautions, the potential convergence issues caused by allele flipping can be mitigated.

4) could you show if the Gtest used for testing the annotation is correctly controlling the type I error

We thank the reviewer for this suggestion. We have added **Supplementary Figure** S3 and **Supplementary Table** S5 as well as lines 136-144 to showcase the accuracy and effectiveness of the proposed G-test:

Specifically, when functional enrichment was simulated (W = 1 and W = 2), the SparsePro G-test identified the enriched annotations as well as annotations that overlapped with the enriched annotations (**Supplementary Table** S5 and **Supplementary Figure** S3). In settings without simulated functional enrichment (W = 0), none of the G-test results were statistically significant (**Supplementary Table** S5 and **Supplementary Figure** S3). Furthermore, even without selecting relevant annotations based on a p-value threshold, the joint enrichment estimates in SparsePro yielded small weights for non-enriched annotations and large weights for enriched annotations, which closely aligned with their simulated values (**Supplementary Table** S6 and **Supplementary Figure** S4).

Reviewer 3:

The authors propose a method for estimating the hyperparameters of the sum of single effects regression. In particular they leverage functional annotation enrichments to specify informative prior inclusion probabilities for different variants, and leverage heritability estimates to set the effect size and residual variance hyper-parameters. By incorporating this enrichment information they are able to demonstrate improvement over fine-mapping methods that either do not leverage functional annotations, or leverage functional annotations through different means (e.g. Polyfun, which computes prior inculsion probabilities given partitioned heritability estimates across a set of annotations). These are important contributions, as the selection of these hyperparameters can greatly influence the calibration and power of finemapping.

We thank the reviewers for their supportive comments.

While I am generally positive about the work put forth here, my main reccomendations are to focus the discussion and commentary on the benefits of including functional annotations, and providing more clear rationale for the use of heritability information to set the effect and error variance hyperparameters. The authors should modify their discussion of the algorithmic differences between SuSiE and SparsePro because they do not seem accurate– as far as I can tell, for a fixed set of hyperparameters (prior inclusion probability, effect variance, residual variance) the coordinate ascent variational inference (CAVI) employed for SparsePro and SuSiE's IBSS algorithm (which is also CAVI) are the same.

We thank the reviewer for the suggestions. We have now expanded our discussion on the benefits of including functional annotations with examples from real data analyses in the **Discussion** section in lines 239-263. We also clarified the algorithmic equivalence in the **Equivalence between the SuSiE IBSS algorithm and a paired mean field variational inference algorithm** section in lines 8-52 of the Supplementary Notes. Additionally, we clarified our approaches for estimating hyperparameters and summarizing posterior probabilities in the **Hyperparameter estimation** section in lines 53-72 of the Supplementary Notes and the **Posterior summaries** section in lines 73-112 of the Supplementary Notes.

Estimating prior inclusion probabilities (Sparspro vs Polyfun) To my understand, Polyfun provides a heuristic for forming the prior inclusion probabilites based on a heritability partition. Sparsepro takes

a less heuristic approach by directly estimating enrichment of selected variants, and using those enrichments to refine the posterior approximations made by SuSiE. I very much support this approach, and the authors successfully show through extensive simulations how their method improves on the heursitic approach to estimating the prior inclusion probabilities developed in Polyfun.

We thank the reviewer for the supportive comments and thorough summary of our work.

Estimating variance hyperparametes (Sparsepro vs SuSiE) SuSiE uses a variational empricial Bayes approach to estimate the effect variance and residual variance– this just means optimizing the objective w.r.t to these hyperparameters. In contrast, Sparsepro fixes these hyperparemeters to values informed by heritability estimates. For example, the residual variance is set to 1-h2. The residual variance is fixed to 1 - h2 where h2 is a locus level heritability estimate. In contrast, a conservative approach would be to set the residual variance to 1. I'm concerned that setting the residual variance to 1-h2 may disrupt calibration of the posterior. Basically, while h2 is an estimate of the heritability in the locus, finemapped association signals will only explain a portion of this heritability. 1-h2 may be too small, and encourage the model to select variants in a way that is anti-conservative.

We thank the reviewer for raising the important point of calibration. We fully agree with the reviewer that fine-mapped signals can only explain a portion of heritability. This is particularly pronounced in calculation of the total trait heritability when the number of variants that are well-powered to be fine-mapped is often overshadowed by the number of variants with small effect sizes. However, in our setting, all variants included for heritability estimation within a locus have also been included in the joint model $\mathbf{y} \sim \mathcal{N}(\mathbf{XS}\boldsymbol{\beta}, \tau_y^{-1}\mathbf{I})$ as described in lines 23-37 of the Supplementary Notes. Thus, variants that are well-powered to be fine-mapped are represented in effect groups that can be summarized into credible sets while variants with small effect sizes are represented by other effect groups. Therefore, we think 1-h2 accurately captures the residual variance unexplained by genetic variants in a locus. As detailed in lines 62-64 of the Supplementary Notes, Shi et al [1] showcased that the HESS estimator accurately and robustly estimate local heritabilities for a variety of genetic architectures. In practice, we also find posterior inclusion probabilities obtained from both SparsePro and SuSiE with our proposed strategies for hyperparameter estimation (SuSiE+HESS) to be well-calibrated as demonstrated in **Figure** 2C and **Supplementary Figure** S8.

The variational approximations are identical The discussion and supplemental materials emphasize the differences in computation between SuSiE's IBSS algorithm, and the variational updates derived in this paper. However, it is important to note that the variational approximation for Sparsepro and SuSiE are identical $q(\beta, S) = \prod_k q(\beta_k, s_k)$. Consequently all differences in performance between SuSiE and Sparsepro- (without annotation) should be explained by (1) differences in the hyperparameters/hyperparameter estimation procedure and (2) implimentation details (e.g. convergence criteria, order of coordinate updates, etc., which may influence which local optima of the variational objective is found). In particular the following does not seem correct 214:215 "In SuSiE, Bayes factors were normalized by sum of Bayes factors across all variants while SparsePro uses the softmax function to normalize posterior probabilities which increased power for identifying causal variants". I believe the marginal log Bayes factors are equal (up to a constant) to the posterior (log) probabilities referenced here (th 4th expression in supplementary material, page 27). Thus normalizing Bayes factors is equivalent to applying softmax of the log probabilities.

We thank the reviewer for pointing out the equivalence. We have now removed the incorrect discussion and clarified the equivalence in the **Equivalence between the SuSiE IBSS algorithm and a paired mean field variational inference algorithm** section in lines 8-52 of the Supplementary Notes.

Suggested Revisions:

- Clarify the similarities and differences between Sparsepro and SuSiE. I believe the variational approximations are the same, but the real contribution here are annotation and heritability informed hyperparameter settings, which are an important contribution that can stand on there own.

We thank the reviewer for this suggestion. We have now clarified our connection with SuSiE in the **Equivalence between the SuSiE IBSS algorithm and a paired mean field variational inference algorithm** section in lines 8-52 of the Supplementary Notes. We also summarized our contributions for estimating hyperparameters and summarizing posterior probabilities in the **Hyperparameter estimation** section in lines 53-72 of the Supplementary Notes and the **Posterior summaries** section in lines 73-112 of the Supplementary Notes. Additionally, we expanded the **Discussion** section in lines 214-234 to clarify our contributions in extending the capabilities of SuSiE.

- Sparsepro- and SuSiE shoud be identical up the the setting of the effect variance and residual variance hyperparameters. Commentary attempting to explain the difference in performance between SuSiE and Sparsepo- should be revised, because at times it implies a difference in the algorithm/optimization procedure which does not seem to be correct.

We thank the reviewer for this comment. We have now removed the incorrect discussion and clarified our connection with SuSiE in the **Equivalence between the SuSiE IBSS algorithm and a paired mean field variational inference algorithm** section in lines 8-52 of the Supplementary Notes.

- Please discuss/justify the heritability based estimates for effect variance and residual variance. In particular I am concerned that useing 1-h2 fro residual variance will make the algorithm anticonservate (see above) by underestimating the residual variance in the regression problem. Assess the calibration of PIPs for Sparsepro+ (e.g. Figure S1 in SuSiE manuscript)– the AUC plots tell us that ranking variants by PIP is good, but it doesn't tell us that thresholding at some nominal PIP value controls the false positive rate. Good PIP callibration would go a long way in addressing my conerns about the choice of residual variance parameter

We thank the reviewer for the suggestion. We have now explained our motivation for estimating hyperparameters with local heritability estimates in the **Hyperparameter estimation** section in lines 53-72 of the Supplementary Notes. Specifically, local heritability estimates can reduce the number of parameters to be estimated by fine-mapping algorithms, resulting in improved accuracy and efficiency. Additionally, we incorporated this strategy in SuSiE and observed improved power for fine-mapping compared to its original implementation. We agree with the reviewer on the importance of calibration. We showed in **Figure** 2C that posterior inclusion probabilities obtained from SparsePro are well-calibrated. We also observed that incorporating local heritability-based hyperparameter estimation in SuSiE can improve power for fine-mapping (**Supplementary Table** S2) while maintaining calibration of posterior inclusion probabilities (**Supplementary Figure** S8) as detailed in lines 65-72 of the Supplementary Notes:

In simulations, we have observed that incorporating local heritability-based hyperparameter estimation can improve power for fine-mapping (**Supplementary Table** S2) while maintaining calibration of PIP (**Supplementary Figure** S8). By applying this strategy to SuSiE, we have observed substantial improve-

ments in performance (**Supplementary Table** S2). For example, in the simulation setting with K = 5 and W = 2, SuSiE+HESS outperformed the original SuSiE by identifying a greater number of true causal variants with higher PIP values (**Supplementary Figure** S11). Moreover, the variant-level PIP obtained from SuSiE+HESS and SparsePro- were highly similar (**Supplementary Figure** S12), both achieving an overall AUPRC of 0.91 (**Supplementary Table** S2).

Minor points

- Maybe a simpler enrichment analysis for the UKBB biomarkers would be (1) causal variants in this phenotype vs (2) causal variants discovered in other phenotypes. It would more clearly highlight that the enrichment of the tissue-specific annotation in the relvant biomarker is above and beyond the background level enrichment of enrichment across causal variants discovered in all phenotypes.

We thank the reviewer for this insightful suggestion. We have now added the suggested enrichment analysis as described in lines 379-381:

Additionally, we examined whether the top variants from 95% credible sets identified for a trait were more enriched for relevant tissue-specific annotations compared to the top variants identified for other traits by Fisher's exact tests.

We presented the results in **Supplementary Tables** S13 and S14, **Figure** 5B as well as in lines 196-199:

Furthermore, the top variants from 95% credible sets also demonstrated tissue specificity (**Figure** 5B). For example, approximately 36% of the top variants for eGFR were annotated to kidney-specific annotations, while only 18% of the top variants for pulse rate were annotated to kidney-specific annotations (p-value from Fisher's exact test: 8.8×10^{-7}) (**Supplementary Tables** S13 and S14).

- Were causal variants defined as the top variant per credible set or all variants in the credible set?

We have now clarified that the causal variants for downstream investigations were the top variant per credible set as in lines 379-381.

Additionally, we examined whether the top variants from 95% credible sets identified for a trait were more enriched for relevant tissue-specific annotations compared to the top variants identified for other traits by Fisher's exact tests.

- It is not clear to me which annotations are used in the UKBB biomarkers analysis. This should be clearly stated in 4.4 or methods.

We apologize for the confusion. We have now clarified in lines 372-374:

Using the summary statistics and the matched LD information [3], we performed genome-wide fine-mapping with “SparsePro-”, “SparsePro+” and “SparsePro+PolyFun” as described in Section 5.3 with annotations from the “baselineLF2.2.UKB” model [3] provided by PolyFun.

- To clarify the comparison between polyfun and Sparsepro it may be good to (1) run Sparsepro with the prior inclusion probabilities derived from polyfun and (2) fit Sparsepro with the exact same annotations used in polyfun (without screening annotations based on significance first). (1) vs Sparsepro+ would demonstrate that Sparsepro+ is making better use of the annotation information. (2) vs Sparsepro+ would emphasize the benfit of selecting annotations based on Gtest.

We thank the reviewer for these important suggestions. We have now added new genome-wide simulations to directly compare “SparsePro+, “SparsePro+PolyFun” and “SparsePro+1.0” (SparsePro without filtering annotations by the G-test) as detailed in lines 344-351:

We applied SparsePro to the GWAS summary statistics with the aforementioned LD information, iterating over all sliding windows initially without any functional annotation. The fine-mapping results obtained were referred to as “SparsePro-”. Next, the 10 annotations used in locus simulations were used to derive functional priors. The fine-mapping results from SparsePro with a prior derived from PolyFun were denoted as “SparsePro+PolyFun”. Additionally, results from SparsePro with a functional prior estimated from annotations with a G-test p-value less than 1×10^{-5} were denoted as “SparsePro+” while results from SparsePro with a functional prior estimated from all 10 annotations were denoted as “SparsePro+1.0”.

We demonstrated in **Figure** 4 that SparsePro+ is indeed making better use of the annotation information, as illustrated in lines 129-154. We also investigated the impact of G-test on fine-mapping results via the comparison between SparsePro+ and SparsePro+1.0. We found that the difference between these two is not dramatic as presented in **Supplementary Tables** S1, S2, S8, S9 and S10, and reasoned in the **Discussion** section in lines 249-255:

However, in SparsePro, filtering annotations by the G-test does not impact the fine-mapping results dramatically (**Supplementary Tables** S2 and S8). This is because when irrelevant annotations are included in the joint estimates, their estimated enrichment weights are typically small (**Supplementary Table** S6), thus having a limited impact on the functionally-informed priors. Nonetheless, in SparsePro, screening annotations by G-test leads to simple interpretable models. While we used a p-value threshold of 1×10^{-5} in both simulations and fine-mapping of functional biomarkers, users can adjust this threshold based on their preference for a more complicated model or a sparser model.

*References

[1] Huwenbo Shi, Gleb Kichaev, and Bogdan Pasaniuc. “Contrasting the genetic architecture of 30 complex traits from summary association data”. *The American Journal of Human Genetics* 99.1 (2016), pp. 139–153.

[2] Michalis Titsias and Miguel Lázaro-Gredilla. “Spike and slab variational inference for multi-task and multiple kernel learning”. *Advances in Neural Information Processing Systems* 24 (2011), pp. 2339–2347.

[3] Omer Weissbrod et al. “Functionally informed fine-mapping and polygenic localization of complex trait heritability”. *Nature Genetics* 52.12 (2020), pp. 1355–1363.

[4] Jian Yang et al. “GCTA: a tool for genome-wide complex trait analysis”. *The American Journal of Human Genetics* 88.1 (2011), pp. 76–82.

[5] Yuxin Zou et al. “Fine-mapping from summary data with the “Sum of Single Effects” model”. *PLOS Genetics* 18.7 (2022), e1010299.
