# Supplementary material for: SparsePro: An efficient fine-mapping method integrating summary statistics and functional annotations
Source: PLoS Genet. 2023 Dec 28;19(12):e1011104. doi: 10.1371/journal.pgen.1011104 (PMC10781022; doi:10.1371/journal.pgen.1011104)
Supplement: Attachment — Submitted filename: Sparsepro_Revision_Review.pdf [file pgen.1011104.s036.pdf]

# Sparsepro Revision Review

The main strength of the proposed methodology are that it
- it provides a way of estimating annotation enrichment and using these enrichments to build an informative prior on which variants are selected.
- advocates for new methodologies for estimating other hyper-parameters in the SuSiE model (residual variant, prior effect variance)
- advocate using new posterior summaries. They approximate posterior inclusion probabilities (PIPs) with a "maximization" based approach and furnish the notion of "attainable credible set" to help prune irrelevant factors in the model.

The value of incorporating annotations and leveraging enriched annotations is well appreciated. The authors do a good job of comparing against PolyFun. The revised manuscript does a much better job empirically demonstrating the improvements made by their hyper-parameter estimation procedure and their proposed posterior summaries. Specifically, I appreciate that they show the improvement in performance achieved by each of these contributions separately and in concert.

The revised manuscript corrects many of the major issues in the first-- namely being unclear/confused about the relationship between the variational approximation used in SuSiE and the one proposed here.

My main comments are that (1) the "effect group" terminology is confusing and (2) I disagree with the explanation for why SparsePro/SuSiE offer computationally efficient inference

**Issue 1: effect group terminology is confusing** -- the language around "effect group" gives the impression that there are multiple variants in each effect group when the model says that there is exactly one non-zero effect per "effect group".

E.g. in the caption for Fig 1: "For the k th effect group, sk is a sparse indicator specifying which variants are included in this effect group and βk represents the corresponding effect size."

But $s_k$ is a one-hot vector, it selects *a singular variant* not *variants*. I appreciate that the approach accounts for uncertainty in *which* variable is selected. think what is meant is that we integrate/average over possible configurations of $s_k$ w.r.t the variational approximation $q(s_k)$, and that the credible set (= the smallest set with achieving some probability under $q(s_k)$ is the "effect group".

Commentary like effect groups "group correlated variables"-- this is the *desired* behavior of the approximate posterior. The model selects one variable per effect group, and we hope that the

approximate posterior concentrates most of the probability on a few correlated variables. To the point, before the heuristic posterior summarization *all variants are in all effect groups* with non-zero probability (what I mean is that $\gamma_{gk}^* \neq 0$). Any "effect group" can be summarized with a credible set-- the summary just may not be very useful if it contains many variants with small probability, or multiple uncorrelated variables.

I suppose I don't have a problem with the term "effect group" per se. You need a name for each "single effect". I would just be more careful with how "effect groups" are characterized in the text. For example Fig 1 the plate labelled "All variants in the k-th effect group" seems odd because all variants are used when updating all effect groups (even if they contribute negligibly to the posterior).

**Issue 2: Confusing description of computation efficiency**

I don't agree with the explanation for why SparsePro and SuSiE are more computationally efficient than an exhaustive search (e.g. CAVIAR). The paper claims that it reduces a search of "variant level configurations" to a search of "effect group level configuration".

219 - 224: "First, it provides an alternative dimensionality reduction perspective on the SuSiE model, shedding light on its computational efficiency by establishing a connection between the SuSiE IBSS algorithm [13, 14] and a well-studied paired mean field variational inference algorithm [18].Specifically, grouping correlated variables into effect groups can greatly reduce the exhaustive variant-level causal configuration searches to effect group-level configuration searches, which leads to a substantial reduction of computation costs."

S16-18 "inferring effect group-level causal configurations is less computationally expensive than exhaustively searching for variant-level causal configurations, leading to significantly improved efficiency."

I don't think this is accurate-- the benefit of the variational approximation is that it avoids explicitly summing over configurations. By making the "effect groups" independent *a posteriori* SuSiE and SparsePro can simply marginalize over the *other effect groups* while updating *this effect group*. Since each "effect group" contains exactly one non-zero effect variable, the problem is easy (that is, not combinatorial).

In short, I don't think it's accurate to say the variational approximation improves computational efficiency through "effect-group level configurations", it does it by avoiding the need to sum over configurations all together. That computational trick depends only on the fact that $q(S) = \prod_k q(s_k)$ which would be true of a "mean field" approximation as well. I think the benefit of the "paired mean field" approximation is that it is the most flexible family that factorizes this way.

**Minor comments**

283: I suggest just defining $\tilde{\pi}$ elementwise.

$$\tilde{\pi}_g = \frac{\exp A_g^T \mathbf{w}}{\sum_{g'} \exp(A_{g'}^T \mathbf{w})}$$

This avoids the need to overload $\exp$ as a scalar operation in the denominator, and an element-wise operation on a vector in the numerator.

I would strike 225-226 "thus providing theoretical guarantee to the accuracy of fine-mapping algorithm". The approximate posterior is indeed multimodal, and empirically provides good approximations. But the paper cited does not make such strong claims on the quality of the approximation.

Figure S13 seems mislabelled-- based on line 110 in the supplement, I expect it to be a comparison of multiplication based PIPs, and the 'maximization-based' PIP and the "multiplication-based" PIP. But the labels are SuSiE+SparsePro vs SuSiE+HESS.

SuSiE vs SuSiE + HESS vs SuSiE + SparsePro: just clarify how the hyper-parameters are set for "SuSiE", e.g. even if you are just running the SuSiE softward with default values are the residual variance and prior variance estimated via empirical Bayes, or fixed to particular values? That is important particularly for readers to understand the difference between SuSiE vs SuSiE+HESS. For example, if the hyper-parameters are fixed, I wonder if we would see the same improvement using the hyper-parameter estimation procedure implemented in the `susieR` package.
