# Supplementary material for: SparsePro: An efficient fine-mapping method integrating summary statistics and functional annotations
Source: PLoS Genet. 2023 Dec 28;19(12):e1011104. doi: 10.1371/journal.pgen.1011104 (PMC10781022; doi:10.1371/journal.pgen.1011104)
Supplement: Attachment — Submitted filename: plos_genetics_second_response_letter.pdf [file pgen.1011104.s037.pdf]

Dear Dr. Zhu,

We thank you and the reviewers for the follow-up suggestions on our manuscript titled ***"SparsePro: an efficient fine-mapping method integrating summary statistics and functional annotations"***.

We have carefully considered the reviewers' comments and incorporated them into our revised manuscript. In the enclosed point-by-point response letter, we have described modifications based on the reviewers' suggestions. We believe that these revisions have significantly improved the quality and clarity of our manuscript and we are confident that the changes address the issues raised by the reviewers.

We thank you again and look forward to hearing from you and *PLOS Genetics* again!

Sincerely,

Wenmin Zhang, on behalf of all authors

Quantitative Life Sciences Program, McGill University

Response to Reviewers

Reviewer 1:

Thanks for addressing the issues. I have the following follow-up questions:

We thank the reviewer for the follow-up questions.

1. SparsePro uses a different formulation, but the underlying model is same as SuSiE. The K effect groups are same as K single effects. So I suggest removing the discussion on 'Equivalence between the SuSiE IBSS algorithm and a paired mean field variational inference algorithm'. The main contributions of the manuscript lie in the functional annotation, hyperparameter estimation, and posterior summary.

We thank the reviewer for the summary of our contributions. We have removed lines 218-225 of the Discussion section.

2. FINEMAP provides credible sets in the output cred file.

We thank the reviewer for the information. However, the credible sets in the output cred files are very different from the credible sets obtained from both SuSiE and SparsePro, and we have not been able to find any relevant description regarding the construction of credible sets from Benner et al. For example, using the latest version of FINEMAP (Version 1.4.2 released on 2023/04/27) with the provided example data, we obtained two cred files with altogether 49 rows. As a result, we are uncertain about how to perform a fair comparison, which may be beyond the scope of this manuscript.

3. In the supplementary note line 82, the authors said 'it might be challenging to find the appropriate threshold' for purity. However, it is important to note that the threshold for entropy (log(20)) is also arbitrary. The highly correlated variants could be more than 50 in complex regions. Does this threshold, 20, correspond to a purity level in your simulations? Could you summarize the purity for the output CSs? How does the result look like if SuSiE uses the corresponding purity filter?

We appreciate the reviewer's comment regarding the challenge of establishing appropriate thresholds for the selection of informative candidate credible sets. However, compared to the purity threshold, the entropy threshold plays a clear and interpretable role in the selection process. Specifically, in the attain-

able coverage-based approach, candidate credible sets are filtered based on their attainable coverages and the entropy threshold is only used in case of no actual causal signals, where the posterior distribution is very similar to the prior distribution, leading to a giant credible set including approximately 95% of all variants fine-mapped. Therefore, it is relatively straightforward to identify the appropriate entropy threshold. For example, in the situation described by the reviewer, if we already know that there could be more than 50 but less than 70 variants in high correlation based on the input LD matrix, we can set the entropy threshold as log70 instead. In practice, we find log20 to be suitable for most loci outside of the HLA region. That being said, the entropy threshold can be omitted if we do not find-map regions without variants demonstrating statistical significance. We have further clarified these differences in lines 98-100 of the Supplementary Notes.

4. I'm still unclear about the CSs around the boundary of central 1MB region. For a 3Mb window, it has 3 parts, left 1Mb, central 1Mb, right 1Mb. The result for the central 1Mb part is used. What about the CS with SNPs at the right end of the central 1Mb and the left end of the right 1Mb? How do you address the results?

We thank the reviewer for the question and apologize for the confusion. Variants in each 3-Mb sliding window were fine-mapped jointly but PIP for all variants located in the 1-Mb region central to the window as well as credible sets with top variants located in the 1-Mb region central to the window were retained. Therefore, if the top variant of a credible set is located in the central 1-Mb region, this credible set will be included in the current window and if the top variant of a credible set is located in the right 1-Mb region, it will be included in the next window. We have revised the text in lines 353-354.

5. Is the τ_β same for all effect groups? SuSiE allows different effect priors for each single effect.

We thank the reviewer for the comment. We appreciate the flexibility of allowing different priors for each single effect in SuSiE. However, we posit that this would increase the number of hyperparameters to be estimated. In SparsePro, we choose to reduce the number of hyperparameters to be estimated. Therefore, we use the same heritability estimate for specifying τ_β. In practice, users can overwrite the HESS estimates by providing the suitable priors with the "–hess" option if there is strong evidence supporting the use of different priors.

6. Extracting information from the large supplementary table S1 according to lines 100-107 is challenging. Consider presenting these results in a figure format or incorporating them into Figure 3 for better clarity.

We thank the reviewer for the suggestion. We have revised the text in lines 100-111 to match the description of Figure 3 and reformatted **Supplementary Table** S1 to include the summary statistics of Figure 3.

7. What's the largest K used when fitting the SparsePro model in simulations and applications? What's the parameter setting for SuSiE, FINEMAP and PAINTOR?

We thank the reviewer for the question. The largest K used for SparsePro, SuSiE and FINEMAP was 10 in both simulations and applications. Due to the high computation cost, PAINTOR only allows up to 3 causal variants per locus. We used the default mcmc mode for PAINTOR- and PAINTOR+ with the "–mcmc" flag and the default stochastic shotgun search mode of FINEMAP with the "–sss" flag and the function "susie_rss" from SuSiE with default settings for SuSiE. For SuSiE+HESS and SuSiE+SparsePro, the local heritability-based estimates for effect size variance and residual variance were provided to "scaled_prior_variance" and "residual_variance" variables in the "susie_rss" function. We have further clarified in lines 318-323 and lines 327-329.

Reviewer 2:

I am positive about publishing this manuscript in PLOS Genetics. I would like to apologize to the authors for having taken a long time before taking the time to read through their revision, I have tried to do it seriously when I had the time to do so. I think the authors answered my concerns, as well as the other reviewers' concerns, in a satisfactory way and put a substantial amount of work into improving the manuscript.

We thank the reviewer for the positive feedback.

Reviewer 3:

The main strength of the proposed methodology are that it

- it provides a way of estimating annotation enrichment and using these enrichments to build an informative prior on which variants are selected.
- advocates for new methodologies for estimating other hyper-parameters in the SuSiE model (residual variant, prior effect variance)
- advocate using new posterior summaries. They approximate posterior inclusion probabilities (PIPs) with a "maximization" based approach and furnish the notion of "attainable credible set" to help prune irrelevant factors in the model.

The value of incorporating annotations and leveraging enriched annotations is well appreciated. The authors do a good job of comparing against PolyFun. The revised manuscript does a much better job empirically demonstrating the improvements made by their hyper-parameter estimation procedure and their proposed posterior summaries. Specifically, I appreciate that they show the improvement in performance achieved by each of these contributions separately and in concert.

The revised manuscript corrects many of the major issues in the first - namely being unclear/confused about the relationship between the variational approximation used in SuSiE and the one proposed here.

We thank the reviewer for the nice summary of our contributions.

My main comments are that (1) the "effect group" terminology is confusing and (2) I disagree with the explanation for why SparsePro/SuSiE offer computationally efficient inference

We thank the reviewer for these comments.

Issue 1: effect group terminology is confusing - the language around "effect group" gives the impression that there are multiple variants in each effect group when the model says that there is exactly one non-zero effect per "effect group". E.g. in the caption for Fig 1: "For the k th effect group, s_k is a sparse indicator specifying which variants are included in this effect group and β_k represents the corresponding effect size." But s_k is a one-hot vector, it selects *a singular variant* not *variants*. I appreciate that

the approach accounts for uncertainty in *which* variable is selected. think what is meant is that we integrate/average over possible configurations of s_k w.r.t the variational approximation $q(s_k)$, and that the credible set (= the smallest set with achieving some probability under $q(s_k)$ is the "effect group".

Commentary like effect groups "group correlated variables"– this is the desired behavior of the approximate posterior. The model selects one variable per effect group, and we hope that the approximate posterior concentrates most of the probability on a few correlated variables. To the point, before the heuristic posterior summarization all variants are in all effect groups with nonzero probability (what I mean is that $\gamma_{kg} \neq 0$). Any "effect group" can be summarized with a credible set– the summary just may not be very useful if it contains many variants with small probability, or multiple uncorrelated variables.

I suppose I don't have a problem with the term "effect group" per se. You need a name for each "single effect". I would just be more careful with how "effect groups" are characterized in the text. For example Fig 1 the plate labelled "All variants in the k-th effect group" seems odd because all variants are used when updating all effect groups (even if they contribute negligibly to the posterior).

We appreciate the reviewer's point on stressing one variable per effect group and we apologize for the confusion around the characterization of effect groups in the text. For better clarity, we have changed the caption for Figure 1 to "s_k is a sparse indicator specifying the variant representation of the kth effect group and β_k represents the effect size of the kth effect group"; modified the labels in the plate model to "All variants"; removed commentary related to the desired behavior of the posterior distribution "grouping correlated variables" in the model description in lines 218-225, lines 13-18 of the Supplementary Notes.

Issue 2: Confusing description of computation efficiency

I don't agree with the explanation for why SparsePro and SuSiE are more computationally efficient than an exhaustive search (e.g. CAVIAR). The paper claims that it reduces a search of "variant level configurations" to a search of "effect group level configuration".

219 - 224: "First, it provides an alternative dimensionality reduction perspective on the SuSiE model, shedding light on its computational efficiency by establishing a connection between the SuSiE IBSS algorithm [13, 14] and a well-studied paired mean field variational inference algorithm [18].Specifically,

grouping correlated variables into effect groups can greatly reduce the exhaustive variant-level causal configuration searches to effect group-level configuration searches, which leads to a substantial reduction of computation costs."

S16-18 "inferring effect group-level causal configurations is less computationally expensive than exhaustively searching for variant-level causal configurations, leading to significantly improved efficiency."

I don't think this is accurate - the benefit of the variational approximation is that it avoids explicitly summing over configurations. By making the "effect groups" independent a posteriori SuSiE and SparsePro can simply marginalize over the other effect groups while updating this effect group. Since each "effect group" contains exactly one non-zero effect variable, the problem is easy (that is, not combinatorial). In short, I don't think it's accurate to say the variational approximation improves computational efficiency through "effect-group level configurations", it does it by avoiding the need to sum over configurations all together. That computational trick depends only on the fact that $q(S) = \prod q(s_k)$ which would be true of a "mean field" approximation as well. I think the benefit of the "paired mean field" approximation is that it is the most flexible family that factorizes this way.

We apologize for the confusion on our description of computation efficiency. We totally agree with the reviewer that the variational approximation improves computational efficiency by avoiding the need to sum over variant-level configurations. What we intended to convey was that if we treated effect groups instead of variants as variables in regression, working with such variables that were independent of each other could be much more efficient compared to working with variants in high LD with each other. We have removed lines 219-224 and lines 16-18 of the Supplementary Notes to avoid confusion.

Minor comments

283: I suggest just defining π elementwise.

$$\tilde{\pi}_g = \frac{\exp(\mathbf{A}_g^T \mathbf{w})}{\sum_{g'=1}^{G} \exp(\mathbf{A}_{g'} \mathbf{w})}$$

This avoids the need to overload exp as a scalar operation in the denominator, and an elementwise operation on a vector in the numerator.

We thank the reviewer for the suggestion. We have revised the definition in line 283.

I would strike 225-226 “thus providing theoretical guarantee to the accuracy of fine-mapping algorithm”. The approximate posterior is indeed multimodal, and empirically provides good approximations. But the paper cited does not make such strong claims on the quality of the approximation.

We thank the reviewer for the suggestion. We have removed lines 225-226.

Figure S13 seems mislabelled– based on line 110 in the supplement, I expect it to be a comparison of multiplication based PIPs, and the “maximization-based’ PIP and the “multiplication-based” PIP. But the labels are SuSiE+SparsePro vs SuSiE+HESS.

We apologize for the confusion. Yes, Figure S13 is the comparison of multiplication-based PIPs and the maximization-based PIPs in the context of summarizing posterior probabilities obtained from SuSiE with local heritability-based hyperparameter estimation. We obtained the same posterior probabilities for both SuSiE+HESS and SuSiE+SparsePro except that in SuSiE+HESS, the default multiplication-based posterior summaries in SuSiE were used while in “SuSiE+SparsePro”, the maximization-based posterior summaries proposed in SparsePro were used. We have further clarified the meaning of these labels in line 330-332, as well as lines 103-105 and lines 110-111 in the Supplementary Notes.

SuSiE vs SuSiE + HESS vs SuSiE + SparsePro: just clarify how the hyper-parameters are set for “SuSiE”, e.g. even if you are just running the SuSiE softward with default values are the residual variance and prior variance estimated via empirical Bayes, or fixed to particular values? That is important particularly for readers to understand the difference between SuSiE vs SuSiE+HESS. For example, if the hyper-parameters are fixed, I wonder if we would see the same improvement using the hyper-parameter estimation procedure implemented in the susieR package.

We thank the reviewer for the comment. Local heritability-based estimates for effect size variance and residual variance were provided to “scaled_prior_variance” and “residual_variance” respectively in both SuSiE+HESS and SuSiE+SparsePro while the default empirical Bayes based hyperparameter estimates were used in SuSiE. We have further clarified the hyperparameter settings in lines 327-329.
